# Supplementary material for: Genetic Diversity of Hepatitis B and C Viruses Revealed by Continuous Surveillance from 2015 to 2021 in Gabon, Central Africa
Source: Microorganisms. 2023 Aug 9;11(8):2046. doi: 10.3390/microorganisms11082046 (PMC10458803; doi:10.3390/microorganisms11082046)
Supplement: Supplementary file 1 [file microorganisms-11-02046-s001.zip › microorganisms-2535852-supplementary.pdf]

Supplementary Materials

Table S1. Primer sequences used in this study.

Name	Sequence
HBV qPCR	
HBV-qPCR_Fw	CCGTCTGTGCCTTCTCATCTG
HBV-qPCR_Rv	AGTCCAAGAGTYCTCTTATGYAAGACCTT
HBV-qPCR_Probe	CCGTGTGCACTTCGCTTCACCTCTGC
HCV qPCR	
HCV-qPCR_Fw	TGCTAGCCGAGTAGYGTTGG
HCV-qPCR_Rv	ACTCGCAAGCACCCTATCAG
HCV-qPCR_Probe	ACCACAAGGCCTTTCGCRAC
HBV genotyping	
HBV_GT_Fw1	GGGTCACCATATTCTTGGG
HBV_GT_Rv1	CCAAAGACAAAAGAAAATTGGTAAC
HBV_GT_Fw2	GAACAAGAGCTACAKCATGGG
HBV_GT_Rv2	CGGTAWAAAGGGACTCAAGATG
HCV genotyping	
HCV_GT_Fw	TATGAYACCCGCTGYTTTGACTC
HCV_GT_Rv	GTAYCTCGTCATAGCYTCCGTGAA
HBV whole-genome sequencing	
HBV_WG1_Fw1	GTGCACTTCGCTTCACCTCT
HBV_WG1_Rv	CCACCTTRTGAGTCCAAGGA
HBV_WG1_Fw2	CTTGAGGCYTACTTCAAAGACTGTG
HBV_WG2_Fw1	GCTCACCTCACCATACAGCA
HBV_WG2_Rv	GAATGCAGGGTCCAACTGAT
HBV_WG2_Fw2	GCTCWCCTCACCATACAGCA
HBV_WG3_Fw1	TCCTGTCCTCCAATTTGTCC
HBV_WG3_Rv	GCCTGAGTGCTGTATGGTGA
HBV_WG3_Fw2	TCAGTGGTTCGTAGGGCTTT

Table S2. HBV strains showing high nucleotide identity to HBV-A3, HBV-A1, and HBV-E strains detected in this study.

HBV-A3: SYMAV-D0231					
#	Description	E value	Identity %	Country	Accession no.
1	HBV isolate O64	0	98.98%	Gabon	AM184125
2	HBV isolate N35	0	98.82%	Gabon	AM184126
3	HBV strain FE-929-MO	0	97.98%	Gabon	EU054331
HBV-A1: SYMAV-H0721					
1	HBV isolate A1-SA	0	98.83%	South Africa	KP234050
2	HBV isolate 833	0	98.55%	South Africa	AY233289
3	HBV isolate rw14-48	0	98.51%	Rwanda	MK512473
HBV-E: SYMAV-H0235					
1	HBV isolate Eafr	0	95.16%	Guinea	KX186584
2	HBV isolate GU1214	0	94.91%	Guinea	GQ161816
3	HBV isolate CAR039	0	94.91%	CAR	AM494693
